# Single Inertial Sensor-Based Neural Networks to Estimate COM-COP Inclination Angle During Walking

**DOI:** 10.3390/s19132974

**Published:** 2019-07-05

**Authors:** Ahnryul Choi, Hyunwoo Jung, Joung Hwan Mun

**Affiliations:** 1 Department of Biomedical Engineering, College of Medical Convergence, Catholic Kwandong University, 24, Beomilro 579beongil, Gangneung, Gangwon 25601, Korea; 2 Department of Bio-Mechatronic Engineering, College of Biotechnology and Bioengineering, Sungkyunkwan University, 2066 Seoburo, Jangan, Suwon, Gyeonggi 16419, Korea

**Keywords:** COM-COP inclination angle, artificial neural network, long-short term memory, inertial measurement unit

## Abstract

A biomechanical understanding of gait stability is needed to reduce falling risk. As a typical parameter, the COM-COP (center of mass–center of pressure) inclination angle (IA) could provide valuable insight into postural control and balance recovery ability. In this study, an artificial neural network (ANN) model was developed to estimate COM-COP IA based on signals using an inertial sensor. Also, we evaluated how different types of ANN and the cutoff frequency of the low-pass filter applied to input signals could affect the accuracy of the model. An inertial measurement unit (IMU) including an accelerometer, gyroscope, and magnetometer sensors was fabricated as a prototype. The COM-COP IA was calculated using a 3D motion analysis system including force plates. In order to predict the COM-COP IA, a feed-forward ANN and long-short term memory (LSTM) network was developed. As a result, the feed-forward ANN showed a relative root-mean-square error (rRMSE) of 15% while the LSTM showed an improved accuracy of 9% rRMSE. Additionally, the LSTM displayed a stable accuracy regardless of the cutoff frequency of the filter applied to the input signals. This study showed that estimating the COM-COP IA was possible with a cheap inertial sensor system. Furthermore, the neural network models in this study can be implemented in systems to monitor the balancing ability of the elderly or patients with impaired balancing ability.

## 1. Introduction

Evaluation of the balance ability of body movement through posture control is a representative area of biomechanics that is studied by many clinicians and rehabilitation engineers [1]. Generally, the risk of fall increases when one encounters irregularly surfaced ground, a sloping path, or when there is an obstacle [2]. In the United States, it has been shown that more than 30% of elderly people above the age of 65 experience a fall at least once a year [3]. Fall may cause serious pain, which may lead to long-term hospitalization [4]. In the elderly, it is known that factors such as lower body muscular atrophy, limitation in range of motion, and cognitive disorder are related to the degradation of balance recovery ability [5]. Therefore, quantitative evaluation of the balancing ability of body posture during walking is needed to improve the biomechanical understanding of factors related to falls.

Trajectories of the center of mass (COM) and center of pressure (COP) are used as representative parameters to evaluate the balancing ability while walking [6]. Joint assessment of both COM and COP (relative position of COM to COP trajectory) can provide a more complete evaluation than interpretation of COM and COP separately [7,8]. A typical parameter is the COM-COP inclination angle (IA) which is defined as the instantaneous orientation of the imaginary line that connects the COM and COP locations [9]. The horizontal distance between COM and COP that can explain a decrease in stability can be quantified using the magnitude of COM-COP IA. Hong et al. [10] used COM-COP IA to investigate the effects of the sloped pathway and age on gait stability. Their results revealed that the elderly show more COM-COP IA alteration in the sagittal plane and increased COM-COP IA velocity change in the single and double support gait phases. This was thought to be a mechanism to compensate for the decrease in balancing ability compared to the young control group. Other similar studies have used COM-COP IA as a parameter to evaluate the balancing ability of patients suffering from Parkinson’s disease [11,12], scoliosis [13], and cerebral palsy [14]. COM-COP IA has also been used to evaluate the balancing ability of pregnant women [15].

The COM-COP IA parameter can only be extracted with a 3D motion analysis system in a specialized space that has fixed force platform devices. This is due to the fact that the COM is calculated based on marker trajectories extracted by optical cameras and anthropometric information (weighted sum of each segment), while the COP is acquired from the force platform [16]. However, a 3D motion analysis system with a time-series synchronized force platform is highly expensive. In addition, it is installed in a limited space [17]. Moreover, “Targeting” problems can arise when one takes unnatural steps onto a force platform, thereby affecting the entire movement of the body [18]. An existing report has suggested that the “Targeting” problem changes ground reaction patterns during walking [19].

These suggested limitations can be easily dealt with by employing a wearable sensor device and artificial neural network (ANN) modeling technique. The most inexpensive and practical wearable sensor device is the inertial measurement unit (IMU), which consists of a three-axis accelerometer, gyroscope, and magnetometer. IMU is widely used in movement analysis [20,21,22]. Generally, sensor signals are smoothed with a low-pass digital filter because the raw signals of the IMU device have thermal-mechanical and electronic noise. Previous studies have suggested various cutoff frequencies to reduce noise during walking. In order to study the walking step time and velocity, the accelerometer signal was filtered at a 2 Hz cutoff frequency [23] while a IMU sensor signal filtered at 25 Hz was used to compute COM trajectories [24]. In addition, the ANN technique can reduce the number of sensors attached to the body and increase the possibility of estimating the COM-COP IA parameter. ANN has a powerful prediction performance when there is no direct physical relationship between input and output. In the field of biomechanics, the ANN provides great solutions when parameters such as electromyogram signals and joint forces do not have a deterministic relationship [25]. Thus, ANN is widely used to predict human activities or to develop biomechanical models [26]. Moreover, the recently suggested long-short term (LSTM) network can provide high accuracy for time series data estimation [27]. Therefore, this technique is presumed to show good performance for estimating changing parameters such as COM-COP IA in a period of walking.

Although the COM-COP IA is a biomechanically useful parameter to evaluate the balancing ability during walking, there has been no reported attempt to extract it using a cheap, wearable IMU sensor. The evaluation of balance control ability using a cheap wearable sensor allows one to monitor the risk of a fall in daily life. Additionally, periodic monitoring and follow-up are needed to assess the effectiveness of rehabilitation following medical treatment [28]. Therefore, development of a simple system, such as a single inertial sensor device, with artificial intelligence technology is important for health-care perspectives. The first objective of this study was to propose ANN models that could estimate the COM-COP IA during walking based on signals using an IMU device attached to the waist. The ANN can show differences in accuracy depending on its own characteristics. Furthermore, the cutoff frequency of the low-pass filter applied to the sensor signal is decided by the high-frequency composition. This can make changes to the input value and affect the performance of the model. Therefore, the second objective of this study was to evaluate how different types of ANN (conventional feed-forward ANN vs. LSTM) and filtering cutoff frequency could affect the accuracy of the model.

## 2. Materials and Methods

### 2.1. Subjects, Apparatus, and Gait Experiments

For this study, we recruited 24 healthy adult males (age: 26.2 ± 1.5 years, height: 171.2 ± 4.3 cm, weight: 67.3 ± 7.1 kg), without any musculoskeletal disorders. The experiments were approved by the local ethics committee and performed at the Biomedical Engineering Laboratory in Sungkyunkwan University, Republic of Korea. All participants provided written informed consent prior to experiments.

**Figure 1:**
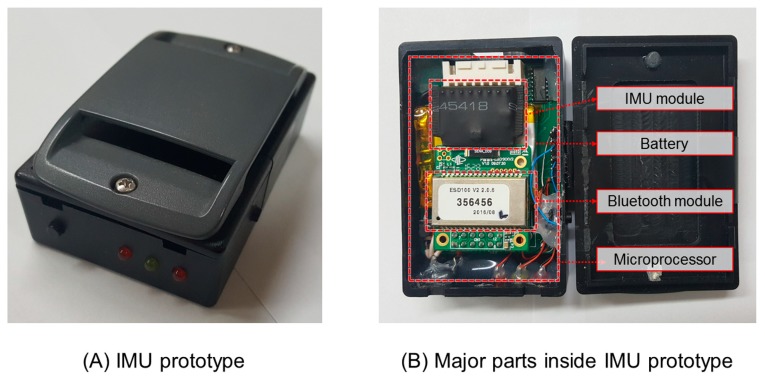
Inertial measurement unit (IMU) prototype.

Six MCam2 cameras (VICON, Oxford Metrics, Oxford, UK) and two OR6-6-2000 force platforms (AMTI Inc., Newton, MA, USA) were used to measure human gait motion. Each system was sampled at 120 and 1080 Hz, respectively. The time-series data acquired from these devices were synchronized using a VICON 460 system. Additionally, an IMU system including an accelerometer, a gyroscope, and a magnetometer was fabricated to estimate the COM-COP IA parameter. The IMU was 45 mm (width) × 70 mm (length) × 25 mm (height) in size. It consisted of an ARM STM 32 microprocessor (AVR, STMicroelectronics, Geneva, Switzerland), an MPU-9250 IMU sensor (InvenSense Inc., San Jose, CA, USA), a battery (3.7 V 2,000 mhA), and a Bluetooth 2.0V module (Chipsen Corp., Gwangmyeong, Korea) (Figure 1). Accelerometer (±16 g), gyroscopic (±2000 deg/s), and magnetic (±4800 uT) signals were sampled at 100 Hz and gathered in the microprocessor. These signals were then transmitted to a PC using a Bluetooth module. The PC interface was implemented using LabVIEW software (National Instruments Corp., Austin, TX, USA). The VICON 460 and IMU system were manually synchronized using major gait events (heel strike and toe-off events) based on a previous study [29].

Subjects were asked to be shirtless with short tight shorts. For each subject, optical markers were attached to 35 anatomical landmarks based on the modified Helen Hays markerset protocol [30]. In addition, the IMU system was positioned on the lower back surface over the 5th vertebra of the lumbar spine [29,31]. Each subject performed a preliminary exercise before participating in the experiment. Each subject also performed a sufficient amount of preliminary gait movements to adapt to the experimental environment. The walking speed was set as normal, fast, and slow. A normal speed indicated a comfortable speed at which the subject normally walks. Fast and slow speeds indicated walking at speeds that the subject considered fast and slow, respectively [32]. Each subject performed five gait experiments.

The marker position and force platform data acquired from the motion capture system during a gait were smoothed utilizing a fourth-order Butterworth digital low-pass filter with a cutoff frequency of 7 Hz [30]. Filtered motion capture data were then used to calculate the COM-COP IA parameter. Additionally, sensor signals of the accelerometer, gyroscope, and magnetometer were filtered using an identical digital filter with three different cutoff frequencies (2, 10, and 25 Hz) in order to evaluate the accuracy of the model. These filtered sensor signals were used as input to develop the artificial neural network models.

### 2.2. Calculation of the COM-COP Inclination Angle

The net COM trajectory of the whole body was calculated utilizing the kinematic method [33]. This method requires an anthropometric model and full kinematic descriptions such as the trajectories of each joint rotation center. In the present study, the human body was divided into 15 segments and 14 joints [34]. The COM for each segment was defined as in a previous publication [35]. The net COP of the entire body was calculated as the weighted sum of trajectories for each COP under the foot, which accounted for the proportion of the vertical ground reaction force for each limb [36].

**Figure 2:**
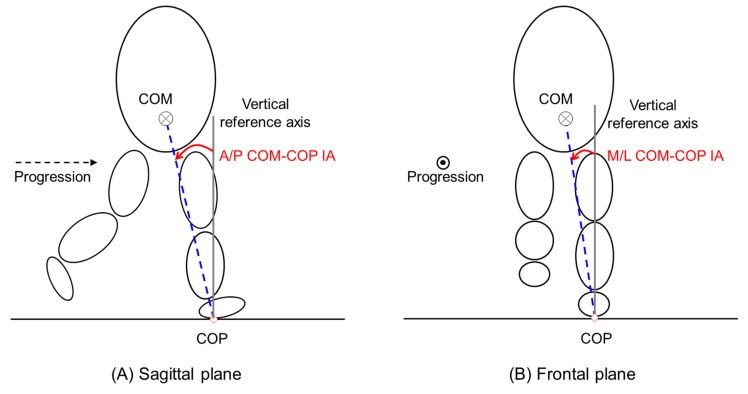
Configuration of the center of mass–center of pressure (COM-COP) inclination angle (IA) in the sagittal plane (**A**) and frontal plane (**B**).

The COM-COP IAs in the sagittal and frontal planes were determined to be the instantaneous orientation of the line connecting the COM and the COP with respect to the vertical line through the COP (Figure 2). They were calculated as follows [16]:

v = (P_COM-COP_ X Z)/‖P_COM-COP_‖ (1)

COM-COP IAs = sin^−1^(v) (2)

where P_COM-COP_ was the vector pointing from the COP to COM, and Z was the vertical unit vector of the global axis. Since two force platforms were set, one gait cycle was defined as the heel-contact point of one foot from the toe-off of the other foot [37]. All data were 100% normalized time-series data.

### 2.3. Artificial Neural Networks

Six different types of neural network models (3 different cutoff frequencies × 2 different models) were proposed in this study. The input vector of each neural network model consisted of 9 time-series signals of 3 axes (anterior/posterior, medial/lateral, and proximal/distal) of the accelerometer, gyroscope, and magnetometer of the IMU device. The input matrix had a size of 36,000 (number of frames: 36,000 = 24 subjects × 5 trials × 3 gait velocities × 100 frames) × 9 (number of input features). Outputs were time-series COM-COP IAs in the sagittal and frontal planes. The output matrix had a size of 36,000 (number of frames) × 2 (number of outputs). Each 9 signal columns of the input matrix and 2 output columns of the output matrix were linearly magnitude scaled between –1 and 1 based on the maximum and minimum values of each column in order to avoid local minima and facilitate the optimization process [25].

**Figure 3:**
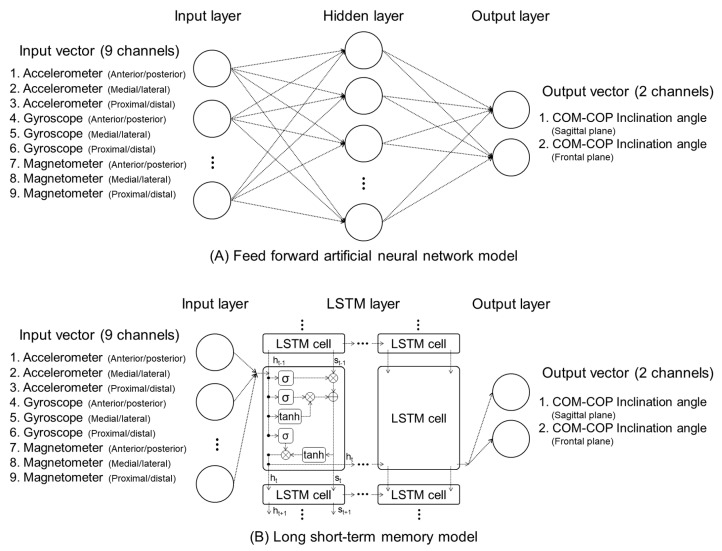
Architectures of the feed-forward artificial neural network (**A**) and the long short-term memory network (**B**).

The feed-forward ANN (FFANN) model consisted of one input layer, one hidden layer, and one output layer (Figure 3A). The number of neurons in the hidden layer was 10. Weight and bias were optimized using a scaled conjugate gradient backpropagation algorithm. The transfer function used the log-sigmoid function between each layer. The maximum number of epochs was set to 1000. However, it was set to stop if the gradient reduction stopped for 6 epochs. The time-dependent deep running model was composed of an LSTM (Figure 3B) using two layers of 512 cells. The state activation function used the hyperbolic tangent function while the optimizer function used the adaptive moment estimation (Adam) stochastic gradient descent method with high convergence performance [31]. The mini-batch size was 10. The learning rate was 0.001. The lambda loss amount was 0.0025, and the epoch number was 100.

Data were divided by the ratio of learning/validation/test as 75/12.5/12.5. Performance was evaluated through a 10-fold cross validation. Data from eighteen and three randomly selected subjects were used for training and validation, respectively, while the three remaining subjects’ data were used for the test. The parameters of the developed FFANN and LSTM network models, such as the number of neurons, transfer function, and learning rate, were determined based on trial and error to minimize the mean-square-error values and to avoid over- or under-fitting [38]. The model implementation was performed using MATLAB R2018b version (The Mathworks, Inc., Natick, MA, USA) and RTX 2080Ti GPU (4352 CUDA cores, 1665 MHz base clock speed, and 11 GB RAM).

### 2.4. Statistics

COM-COP IA values calculated using the 3D motion analysis system and predicted using the proposed model were compared based on the correlation coefficient, root-mean-square error (RMSE), and relative RMSE (rRMSE) values [30]. ANOVA was used to compare the accuracy between conventional FFANN and LSTM models and between cutoff frequencies. The significance level was set to be *p* < 0.05 or *p* < 0.01. All statistical analyses were performed using PASW Statistics 18 (Ver. 18, SPSS Inc. Chicago, IL, USA).

## 3. Results

**Figure 4:**
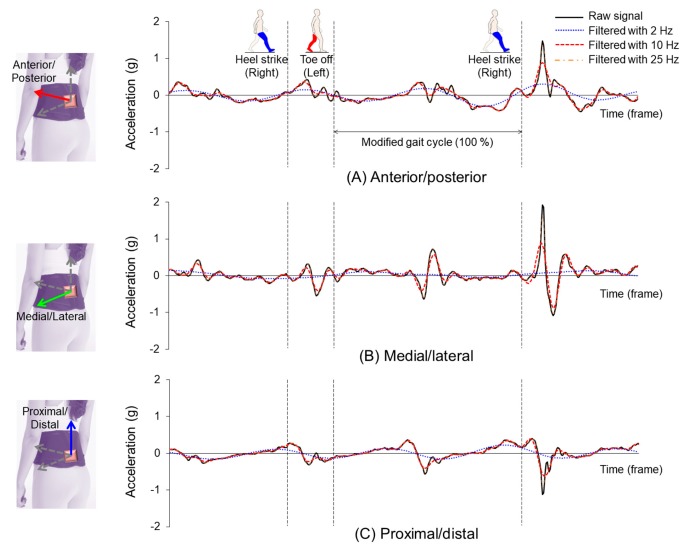
Representative three-axis acceleration signal acquired from the IMU device attached to the waist region.

Figure 4 demonstrates representative raw and filtered three-axis acceleration signals during walking. For the raw signals, no constant value or signal pattern appeared at the time of the gait event and one cycle. The signal using a cutoff frequency at 25 Hz was similar to the original signal with a slight difference in peak point. In contrast, in the signal with cutoff frequency at 2 Hz, the high-frequency component was removed, and a typical acceleration signal pattern appeared in all directions.

**Figure 5:**
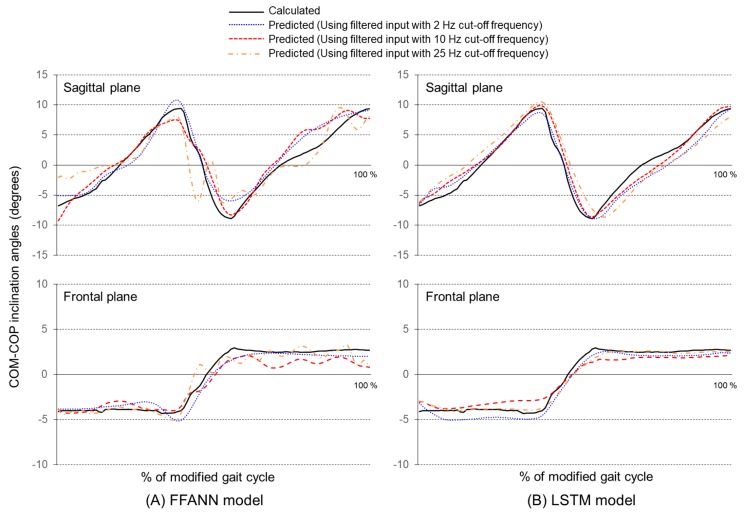
COM-COP inclination angle (IA) calculated using the 3D motion analysis system and predicted using the proposed neural network models.

Figure 5 shows the COM-COP IAs calculated from the 3D motion analysis system and predicted from the proposed models during a modified gait cycle. Changes in the COM-COP IA value on the sagittal plane occurred within 20°, while such changes on the frontal plane occurred within 10°, with half of those on the sagittal plane. In the FFANN model, the pattern of the COM-COP IA value was fairly well-matched compared with the measured value. However, when the filtering of the cutoff frequency of the input signals increased, the predicted values of COM-COP IA on both the sagittal and frontal planes exhibited large upward and downward wave phenomena (Figure 5A). On the other hand, predicted COM-COP IA values from the LSTM model demonstrated good correspondence with the calculated ones, regardless of the filtering cutoff frequencies of the input signals (Figure 5B).

Table 1 shows the correlation coefficients and RMSE values extracted through 10 cross validation experiments. The FFANN model showed correlation coefficients of 0.73–0.81 and RMSE values of 3.01–3.76 in the sagittal plane and correlation coefficients of 0.84–0.87 and RMSE values of 1.27–1.42 in the frontal plane. For the LSTM model, correlation coefficients of both the sagittal and frontal planes were 0.9 or more, higher than those for the FFANN model. In addition, the RMSE values were 1.97–2.24 on the sagittal plane and 0.82–0.85 on the frontal plane, showing improvement compared with the FFANN model.

**Figure 6:**
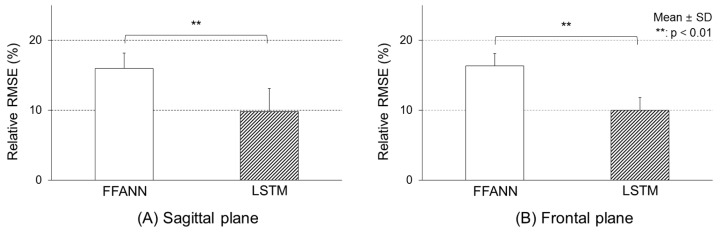
Relative RMSE values between the calculated and predicted COM-COP IAs.

Figure 6 shows quantitative comparisons of accuracy between the FFANN and LSTM models using relative RMSE values. The predicted accuracy of the sagittal plane COM-COP IA was about 16 (2.1)% for the FFANN model and 9.9 (3.2)% for the LSTM model (F[1, 58] = 81.6, *p* < 0.01). For the frontal plane, it was about 16.3 (1.8)% for the FFANN model and about 10.0 (1.8)% for the LSTM model (F[1, 58] = 207.1, *p* < 0.01).

**Figure 7:**
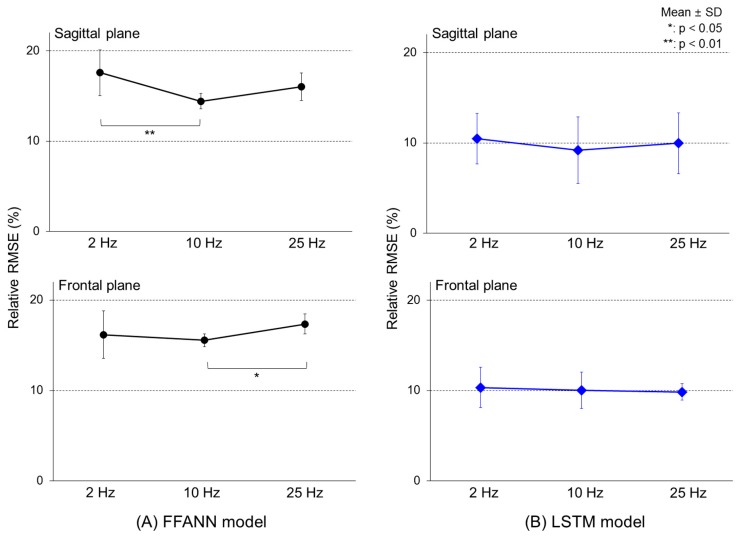
Relative RMSE values between the calculated and predicted COM-COP IA with different cutoff frequencies.

The relative RMSE values between the calculated and predicted COM-COP IAs with different cutoff frequencies of input signals were different in the FFANN model (Figure 7A). There were significant differences among the three cutoff frequencies in the sagittal plane (F[2, 27] = 8.58, *p* < 0.01) and the frontal plane (F[2, 27] = 3.34, *p* < 0.04). In the sagittal plane, the model using the 10 Hz cutoff frequency showed a rRMSE of 14.4 (0.9)%, about 3.1% smaller than with a cutoff frequency of 2 Hz (*p* < 0.01), while the 10 Hz cutoff frequency model on the frontal plane showed an rRMSE of approximately 1.8% smaller than that of the 25 Hz model (*p* < 0.05). However, the LSTM model showed rRMSE values of 10.5 (2.7), 9.2 (3.7), and 10.0 (3.4) on the sagittal plane and 10.3 (2.2), 10.0 (2.0), and 9.8 (1.0) on the frontal plane for the 2, 10, and 25 Hz models, respectively (Figure 7B). In the LSTM model, both the sagittal (F[2, 27] = 0.41, *p* = 0.67) and frontal planes (F[2, 27] = 0.20, *p* = 0.81) showed similar rRMSE values to the cutoff frequency change.

## 4. Discussion

ANNs have been used in various fields and have shown good performance, especially in the fields of medicine and biomedical engineering [39]. Kipp et al. (2018) estimated articular joint torque using only weight and trajectory information of the barbell during weight-lifting [40]. The coefficient of determination between calculated and predicted values was 0.79 to 0.95, and the relative error was 5% to 16%. In addition, Ngoh et al. (2018) conducted a study using IMU sensor signals to predict the vertical ground reaction force using acceleration information in humans running, and they showed high accuracy with a correlation coefficient of 0.99 and an RMSE of 0.017 (N/BW) [20]. The results of the LSTM model of the present study showed that the correlation coefficient was 0.90–0.96 and the rRMSE accuracy was about 10%, similar to those of previous studies (Table 1). Liu et al. [41] concluded that the model is highly accurate if the correlation coefficient is 0.9 or more and the rRMSE is 15% or less, although their studies are limited to artificial neural network studies predicting joint torque. Therefore, the performance of the model in this study is considered to be excellent, although it is rather difficult to directly compare the performance of the neural network model, because various parameters, including the input and output and training data, are different.

In the present study, two types of artificial neural networks (FFANN vs. LSTM) were developed and compared. Comparing the prediction accuracy of COM-COP IA values between the sagittal and coronal planes, the correlation coefficients were about 0.14, and the error was reduced by about 8% (Figure 5 & Table 1). These results show that the LSTM neural network can influence the output of the LSTM neural network by inputting data at the previous time point, unlike the conventional FFANN model which predicts the present time point data with the present data only. LSTM, a kind of recurrent neural network, is composed of a composite network that uses both an output of t-1 and an input of t to output the result of t in time series information [42]. Therefore, time-series based output information can be estimated continuously at every time point. Similar results were obtained in the present study. The FFANN model showed that the high-frequency component of the input was present as the output (Figure 4A). In the case of LSTM, the effect of the high-frequency component was canceled out in the training process with the result of the previous output (Figure 4B).

In addition, the performance of the model was analyzed by changing the cutoff frequency of the low-pass filter to eliminate signal noise. In general, for human walking, the analysis of the harmonic motion signal of each joint’s gesture showed the highest frequency in the forefoot and heel, and 99.7% of total signals were distributed below 7 Hz [43]. In IMU-based walking studies, various cutoff frequencies from 2 to 25 Hz have been applied [23,24]. Actually, if a machine learning model is constructed by inputting nine sensor data extracted from the IMU, the output value may be influenced, because the sensor value is changed according to the cutoff frequency. If the cutoff frequency is too small and the original signal is smoothed as well, the learning of the model may not be efficient (Figure 6A). In the FFANN model of this study, the high-frequency component of the sensor signal according to the cutoff frequency (Figure 3) affected the training of the model. Thus, the high-frequency component was reflected directly in the prediction result (Figure 4A). Nevertheless, the LSTM model was less affected by the cutoff frequency variation. It exhibited a stable performance (Figure 6B). Therefore, when using the FFANN model, an appropriate cutoff frequency should be considered. If the influence of the high-frequency component on the output remains, an additional high-frequency filtering process or data smoothing technique will be needed.

The proposed neural network models in this study had an architecture to predict the output (COM-COPIAs) of the current framework from the input (sensor signals) at a given frame step. Many previous studies have developed machine learning models for the classification of fall or activities of daily life using sensor signals with a proper window size [44]. Using time-window strategies allowed us to increase the dimension of input variables, leading to an increased possibility of model performance through feature extraction. However, there are several limitations associated with increased computational costs and latency of detection [45]. Because the accuracy of the proposed models was fairly high with correlations coefficient above 0.9 without any time-window strategies, a cost-effective and real-time system can be implemented at the commercialization stage as future work.

This study has the following limitations. First, the model was trained with only normal walking data, while data from patients with assorted diseases were not applied. However, this study is the first attempt to suggest whether the neural network and three sensor signals extracted from the IMU can be used to estimate the COM-COP IA parameter during walking. Further studies are needed to reinforce the usability of this model using walking data from the elderly and patients with impaired balancing ability. The second limitation was that the model’s performance was analyzed based on three fixed cutoff frequencies, while other diverse cutoff frequencies were not considered. Yet, the main purpose of this research was to determine whether the cutoff frequency could affect the model’s performance. Further research is needed to find the cutoff frequency that can maximize the model accuracy via performance analysis or an optimization technique. Third, noise control due to the potential magnetic field interference was not addressed in this study. Although these signals were used to train neural network models, the prediction accuracy of LSTM network was fairly high with a correlation coefficient above 0.9. In future studies, greater attention to the quality of the raw signals of the magnetometer is needed to improve the prediction ability of the model. Fourth, the time-series signals acquired from VICON and IMU system were manually synchronized using heel strike and toe-off events during walking, while synchronization error was not addressed in this study. Further study is needed to better improve the accuracy and precision of the prediction models by using a real-time operating system of integration systems. Last, the number of machine learning techniques was limited. This study developed a conventional FFANN model and a LSTM model that performed strongly on time-series data. The use of other various machine learning techniques such as random forest and the support vector machine (SVM) will further improve the performance of the model.

## 5. Conclusions

This study suggests that artificial neural networks can estimate the COM-COP IA parameter during walking using a wearable IMU attached to the waist. The accuracy of the proposed conventional FFANN model was compared to that of LSTM. It was also compared between filtering cutoff frequencies of 2 vs. 10 vs. 25 Hz applied to input signals. The FFANN model showed a correlation coefficient of 0.73–0.86 with an rRMSE of 15%, while the LSTM model showed a correlation coefficient of 0.90–0.96 and an rRMSE of 9%, showing an improved accuracy. For the FFANN model, the best result was obtained when the cutoff frequency of 10 Hz was applied to the input signals, showing a correlation coefficient of 0.84 and an rRMSE value of 14%, while the LSTM model displayed a stable accuracy regardless of the cutoff frequency of the input signal. This study shows that estimating the COM-COP IA parameter is possible with cheap, wearable IMU equipment. Further, the results of this study could be implemented in systems to monitor the balancing ability of the elderly or patients with impaired balancing ability.

## Figures and Tables

**Table 1:** Coefficient of correlation and root-mean-square error (RMSE) values between COM-COP IA calculated using a 3D motion analysis system and predicted using neural network models. FFAN: feed-forward ANN; LSTM: long-short term model.

	Filtering Cutoff Frequencies of Inputs	FFANN		LSTM	
		r	RMSE (deg)	r	RMSE (deg)
Sagittal plane	2 Hz	0.73	3.76 (0.54)	0.90	2.24 (0.61)
	10 Hz	0.81	3.01 (0.18)	0.92	1.97 (0.81)
	25 Hz	0.76	3.43 (0.32)	0.91	2.13 (0.71)
Frontal plane	2 Hz	0.86	1.33 (0.22)	0.95	0.85 (0.19)
	10 Hz	0.87	1.27 (0.05)	0.96	0.82 (0.16)
	25 Hz	0.84	1.42 (0.10)	0.96	0.81 (0.10)
